# The immune response and protective efficacy of a potential DNA vaccine against virulent *Pasteurella multocida*

**DOI:** 10.1186/s43141-021-00180-9

**Published:** 2021-05-31

**Authors:** Ahmed A. M. Yassein, Ayaat A. Teleb, Gamal M. Hassan, Zaki A. El Fiky

**Affiliations:** Genetics Department, Faculty of Agriculture, Fayoum University, 63514 Fayoum, Egypt

**Keywords:** *OmpH* gene, PCR-RFLP, pUCP24-*OmpH*, DNA vaccine, *Pasteurella multocida* serotype a

## Abstract

**Background:**

*Pasteurella multocida* is the main cause of several infections of farm animals, and the immunity gained from commercial vaccines is for the short term only and needs to be routinely administered, so work on new vaccines against virulent *P. multocida* is crucial.

**Results:**

In this study, the *OmpH* gene was amplified from ten *P. multocida* strains, and the PCR products were sequenced and analyzed. The results of RFLP analysis of *OmpH* gene digested by *MspI* enzyme showed that all of ten strains examined possessed one restriction site and two fragments, 350 and 650 bp. The *OmpH* sequence of strain No. 10 was cloned into bacterial expression vector pUCP24, and the recombinant pUCP24-OmpH was expressed in *E. coli* DH5α. Serum samples obtained from the ELISA test from a group of vaccinated rats indicate that the antibodies were present at high titer in immunized rats and can be tested as a vaccine candidate with a challenge.

**Conclusions:**

In rats infected with the DNA vaccine and inactivated vaccine, a significant increase in serum antibody levels was observed. In addition, the DNA vaccine provided the vaccinated rats with partial protection; however, the protective efficacy was greater than that offered by the live attenuated vaccine. This successful recombinant vaccine is immunogenic and may potentially be used as a vaccine in the future.

## Background

Livestock owners and crevice animals vaccinated with inactivated or destroyed vaccines have appropriate questions as to the extent of site reactions to the injection, with justified concerns as to the severity of injection site reactions. However, these ideas may become worries of the past, since DNA vaccines are not formulated with chemical adjuvants, the leading contributor to injection-site reactions. Since DNA vaccines consist simply of nucleic acids, they lack the ability to replicate, infect, or induce disease [1].

DNA vaccination, or genetic vaccination, is the common name for methods of vaccination that cause immunity by transfecting DNA encoding host cells with an antigen, rather than by injecting protein or peptide antigens. The immune response resulted from DNA vacination in animal host transfected cells was similar to traditional vaccines [2], it has been shown that DNA vaccines effectively bypass maternal antibody involvement [3] and that no anti-DNA antibodies are found [4]. A large number of experimental DNA-based veterinary vaccines have been evaluated in a number of species with varying degrees of immunological response and efficacy against challenge [5, 6].

Pathogenic strains of *P. multocida* allow the virulence-associated genes to expression under host conditions. Many of these genes, particularly the OMP-encoding genes, have been extensively studied, and some have been used as the basis for the creation of vaccines that can heterologically protect against multiple strains of *P. multocida* from infection [7–9]. Some studies have shown that some outer membrane proteins (OMPs) of *P. multocida* have contributed to pathogenesis and possess immunogenic and bactericidal properties [10–14]. *OmpH*, a porin, is a general transporter that allows diffusion of various molecules simpler [15]. Another research identified associations between *OmpH* alleles and capsular types of 83 *P. multocida* isolates, and found variable *OmpH* alleles within capsular type A isolates [16]. *OmpH* is an antigenic, surface-exposed, and preserved OMP porin found in 100% of the bovine isolates investigated and mooted as a possible candidate for a vaccine [17].

The *P. multocida* toxin (PMT) DNA vaccine has the potential to protect infected animals from infections caused by *P. multocida* [18], while this DNA vaccine was able to provide protection against avian pasteurellosis by using two separate OMPs, *OmpH* and *OmpA*, and showed the highest degree of protection [19, 20]. The pVAX1-ABA392 DNA vaccine is capable of producing a high titer of anti-HS antibodies caused by *P. multocida* and has the potential to be a vaccine candidate [21]. The aim of this study was therefore to characterize the nucleotide sequence of the *OmpH* gene using molecular genetics and bioinformatics techniques. In addition, a potential pUCP24 OmpH DNA vaccine should be developed and its protective efficacy evaluated.

## Methods

### Bacterial strains and expression vector

*Pasteurella multocida* strains used in this study were isolated and identified in previous study in our laboratory [22]. *Escherichia coli* DH5α strain was obtained from Dr. Mercedes, Facultat de Farmacia, Barcelona, Spain. The expression vector pUCP24 was obtained from Novagen, (Germany) for cloning and gene expression.

### Amplification of *OmpH* gene and PCR–RFLP

Genomic DNA from ten strains of *P. multocida* was isolated using the XS method [23]. Primers for amplification of major outer membrane protein H (*OmpH*) gene of *P. multocida* strains were designed according to the nucleotide sequences of the *OmpH* gene of *P*. *multocida* pm70 (NCBI accession number NC_002663). The *OmpH gene* was amplified using gene-specific oligonucleotide primers: OmpH-KpnI forward TGAGGTACCATGAAAAAGACAATCGTAG and OmpH-EcoR1 reverse TAGGAATTCTTAGAAGTGTACGCGTAAAC. The PCR-amplified products were analyzed on 1.2% agarose gel and purified using a protocol described by Li et al [24]. With regard to RFLP banding patterns, each of PCR products (*OmpH* gene) was digested using BglII or MspI restriction enzymes (Thermo Scientific™, ER0081 and ER0541, respectively), according to the manufacturer’s instructions. Fragments were then analyzed in 2% agarose on 10% TBE under UV illumination. A molecular weight marker (1kb DNA ladder, Fermentus) was added for each of the gel runs.

### Sequencing of *OmpH* gene and phylogenetic analysis

The purified *OmpH* gene PCR products were sequenced using an API 3730X1 Automated DNA sequencer (Applied Biosystems, USA). The sequence data were analyzed using the National Center for Biotechnology Information (NCBI) database using the BLAST. *OmpH* gene sequences from strain Nos. 2, 5, 7, and 10 were subjected to alignment with the sequences of *P. multocida* in the GenBank sequence database using the BLAST in program. Phylogenetic analyses were conducted according to Tamura et al. [25]. The MEGA 4 program was used to generate a phylogenetic tree using the UPGMA method.

### Construction of recombinant plasmids

Each of *OmpH* gene PCR products and pUCP2 expression vector was digested using *Kpn1*/*EcoR1* restriction enzymes, (Thermo Scientific^Tm^Fermentas, Germany, ER0521 and ER0271, respectively), according to the manufacturer’s instructions. After purification, they were ligated in ligation mixture consisting of 5 μl deionized water, 1 μl pUCP24 expression vector, 2.5 μl PCR product, 1 μl T_4_ ligase buffer, and half microliter of T_4_ ligase enzyme. Then, they were kept in a microfuge tube and spun for a few seconds. The vial was incubated at 22 °C for 2 h; then, the ligation mixture was ready for transformation.

### Transformation of *E.coli* competent cells

Competent cells of *E. coli* DH5α were transformed using rapid transformation procedure (Life Technologies Corporation 2013) as user guide: *E.coli* competent cells were taken from a −80 °C freezer in 1.5-ml tube and kept on ice. Five microliters containing 50 ng of recombinant plasmid DNA (circular) was added to 50 μl competent cells and incubated on ice for 10 min. Tubes were transferred to water bath at 42 °C for 45 s and then back on ice for 2 min to reduce damage to the cells. One milliliter of LB antibiotic-free medium was added, and tubes were incubated for 1 h at 37°C in a shaking water bath (200rpm). One hundred microliters of the resulting culture was spread on LB plates with antibiotic (ampicillin) and colonies picked after about 12–16 h.

### Vaccination protocol

Forty male albino rats weighing 90–110 g (8–10 weeks old) were obtained from the Egyptian Company for Production of Vaccines, Sera & Drugs (EGYVAC), Helwan, Egypt. Rats were housed in universal polypropylene cages and kept in the animal house of the Biochemistry Department, Faculty of Agriculture, at 25±2°C with 12 h of continuous fluorescent lighting. Animals fed a standard synthetic diet were obtained from Feedmix Company in Cairo and had free access of tap water, ad libitum. Rats were kept for 1 week for adaptation, before starting the experiment. Rats were divided into four groups equal in weight (ten rats each). The rats were assigned randomly to four groups, i.e., (1) the control group, (2) inactivated vaccine group, (3) the DNA vaccine (pUCP24-*OmpH*) group, and (4) the pUCP24 group. The rats in the control group were injected with 100 μl PBS (0.01 M, pH 7.2), the rats in group 2 were injected with 100 μl inactivated vaccine, the rats in group 3 were injected with 100μg/100μl of recombinant plasmids isolated from transformed bacteria, and the rats in group 4 were injected with 100 μg/100 μl of vector (pUCP24).

### Detection of serum antibody levels

After immunization, blood samples were drawn from the rats, and serum samples were collected from the immunized rats at 0-, 14-, and 28-day post-vaccination. The serum antibody titers were tested using indirect enzyme-linked immunosorbent assay (ELISA) [26].

### Challenge study

Each group of experimental containing ten apparently healthy rats were challenged by inoculating intraperitoneally after 10 days of the 2nd DNA immunization with 0.1 ml (10^6^ CFU) of virulent *P. multocida* type A, and returned to their cages. Rats were fed with pelleted feed and water ad libitum before and during the experiment. All experimental rats were kept under the same environment. Clinical and postmortem examination was conducted on any rat that died, and the lungs were collected for histopathological tests and re-isolation of *P. multocida.* The survival number and protection rate were counted. Diethyl ether was used for animal anesthesia before samples were taken for histopathological studies.

## Results

### Amplification and RFLP of *OmpH* gene

**Fig. 1:**
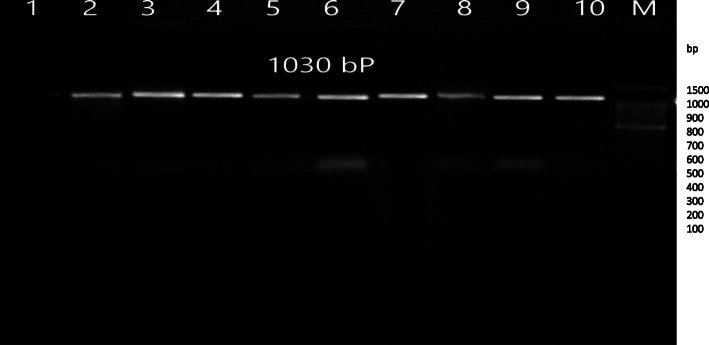
Agarose gel electrophoresis of ten strains of *OmpH* gene of *Pasteurella multocida*. Lanes 1–10 PCR product and M 100 bp DNA ladder

**Fig. 2:**
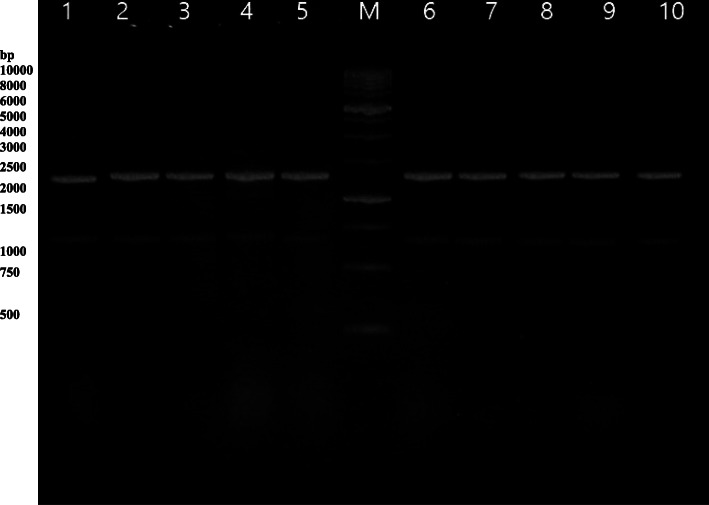
RFLP patterns of *OmpH* DNA after digestion with restriction enzyme *Msp*I. Lanes 1–10 PCR product and M 1kbp DNA ladder

**Fig. 3:**
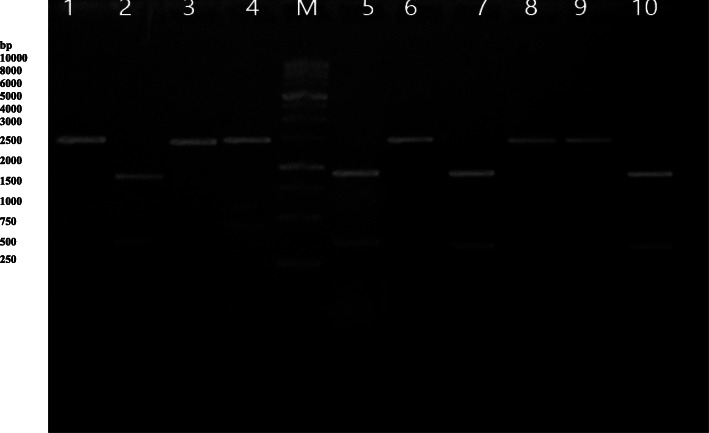
RFLP patterns of *OmpH* DNA after digestion with restriction enzyme *Bgl*II. Lanes 1–10 PCR product and M 1kbp DNA ladder

The target gene fragments were amplified using the genomic DNA of ten *P. multocida* strains as a template. PCR amplification of the *OmpH* gene yielded the expected product of 1030 bp. A single PCR product with a similar molecular size was obtained from each strain (Fig. 1). The results of RFLP analysis of *OmpH* gene digested by *MspI* and *Bgl*II restriction enzymes are presented in Figs. 2 and 3, respectively. Digestion of *OmpH* gene with *MspI* enzyme showed that all of the ten strains examined possessed one restriction site and two fragments, 350 and 650 bp (Fig. 2). Whereas *Bgl*II enzyme digestion of *OmpH* gene divided the strains into two groups, four out of ten strains showed a restriction site producing two fragments, 250 and 800bp, and the rest of the strains had one band, 1000 bp (Fig. 3).

### Sequencing of *OmpH* gene and phylogenetic analysis

**Fig. 4:**
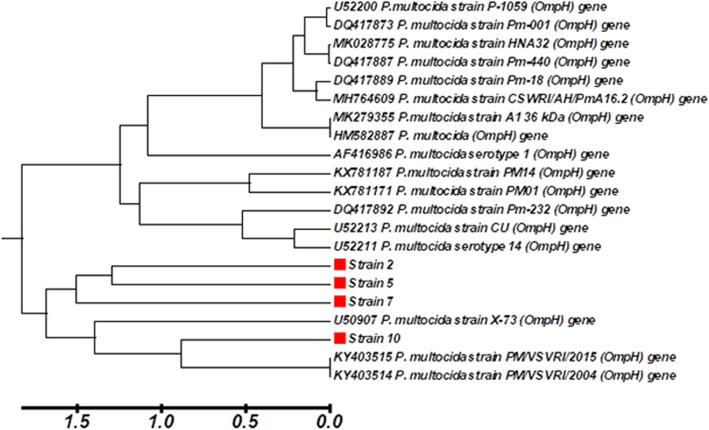
Neighbor-joining phylogenetic tree of *OmpH* gene from *P. multocida* strain Nos. 2, 5, 7, and 10 with different nucleotide sequences of *Pasteurella multocida OmpH* gene

Single-fragment nucleotide sequences from each PCR product of *OmpH* gene amplified from strain Nos. 2, 5, 7, and 10 were sequenced and analyzed using DNASTAR (DNASTAR Inc., Madison, WI) and MEGA4 programs [25]. The multiple sequence alignment reveals the high identity of these four *OmpH* sequences to other *P. multocida OmpH* sequences in the GenBank database. The topology of the neighbor-joining tree represented a monophyletic group (Fig. 4). The phylogenetic tree revealed that these four sequences were closed related to the *P. multocida OmpH* gene sequences obtained fron GenBank. The tree was successfully grouped into two main clusters. The *OmpH* DNA sequences of strain Nos. 2, 5, 7, and 10 closed in the first cluster with three *P. multocida* strain serotype A: U50907 strain X-73, KY403515 strain PM/VSVRI/2015, and KY403514 strain PM/VSVRI/2004. Moreover, strain No. 10 was closed with the latest two strains. In addition, the second cluster consisted of closed 14 GenBank accessions (Fig. 4).

### Confirmation on recombinant clone

**Fig. 5:**
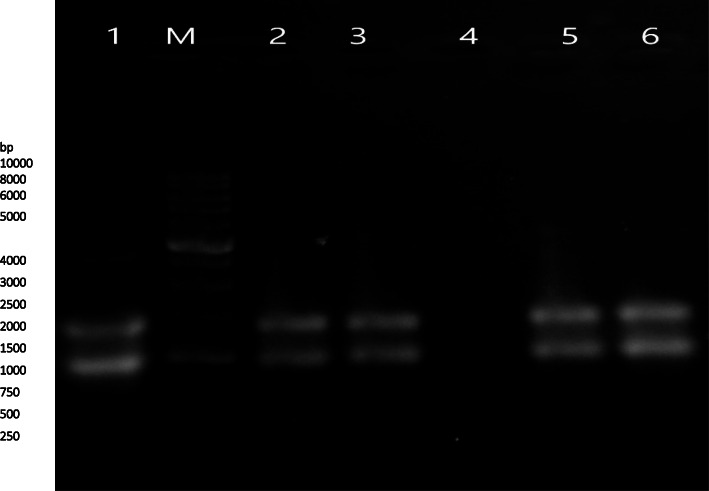
Agarose gel electrophoresis for plasmid DNA purified from recombinant clones of *E. coli* DH5α. Lane 1 pUCP24 vector; M 1kbp DNA ladder; lanes 2, 3, 5, and 6 recombinant plasmid (pUCP24-*OmpH*) for strains 3, 4, 6, and 7 respectively; and lane 4 negative control

The purified PCR products of the OmpH-EcoR1/Kpn1 gene were cloned into the pUCP24 vector digested with EcoR1/Kpn1. The mixture of ligation was then transformed into competent *E. coli* DH5α. The recombinant clones of *OmpH* gene into pUCP24 vector were successfully carried out. The recombinant pUCP24-OmpH plasmid was confirmed via agarose gel electrophoresis as shown in Fig. 5, and affirmed the size of the pUCP24-OmpH recombinant plasmid at 5000bp meanwhile the non-insert vector size at 4035bp. These recombinant plasmids were used to study the development of DNA vaccine and its protective efficacy.

### Immunogenicity determination and challenge

**Fig. 6:**
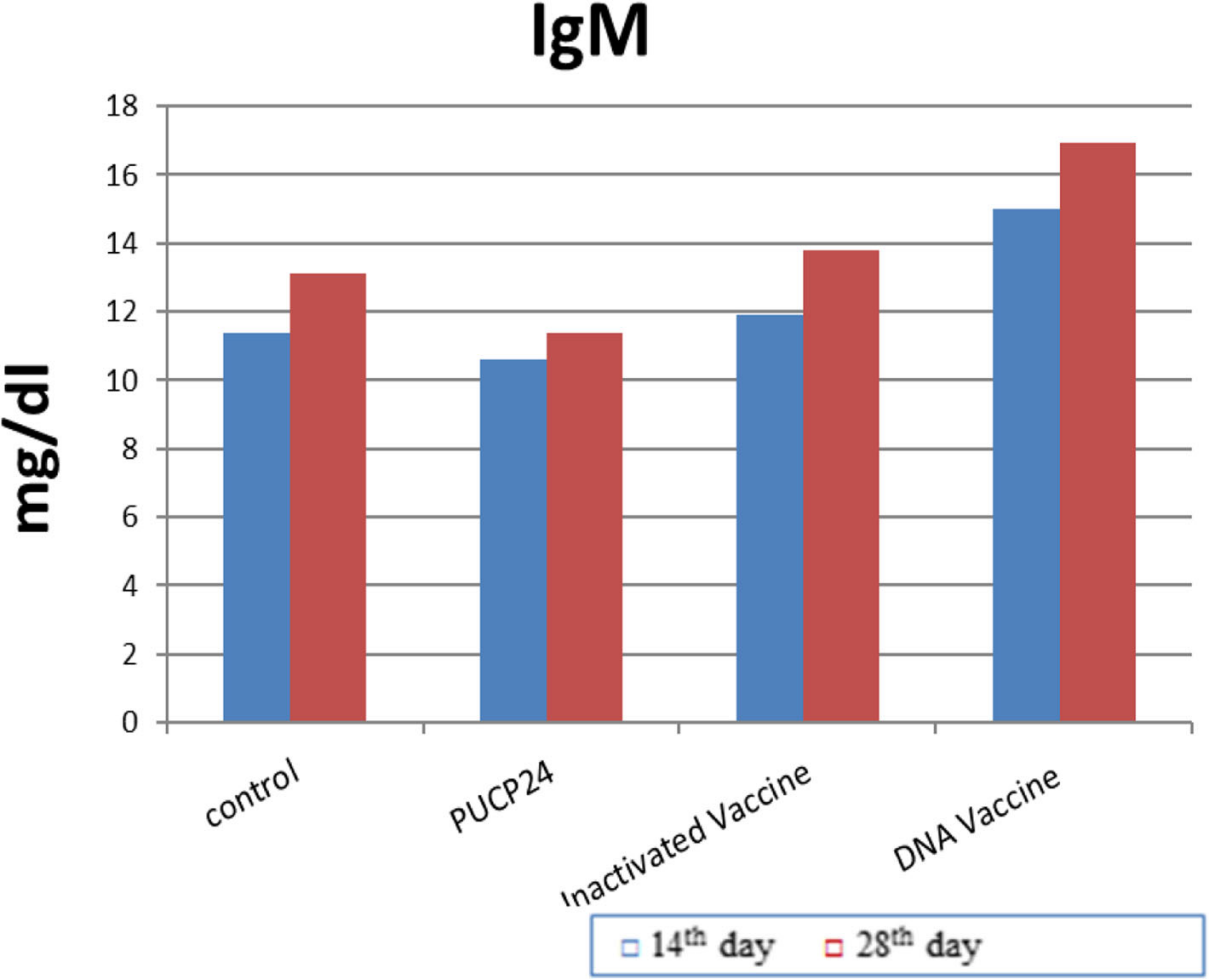
Serum IgM responses in immunized rats measured by indirect ELISA at the 14th day and 28th day of post-immunization. Each bar represents the mean O.D. ± standard error of five pooled serum samples

**Fig. 7:**
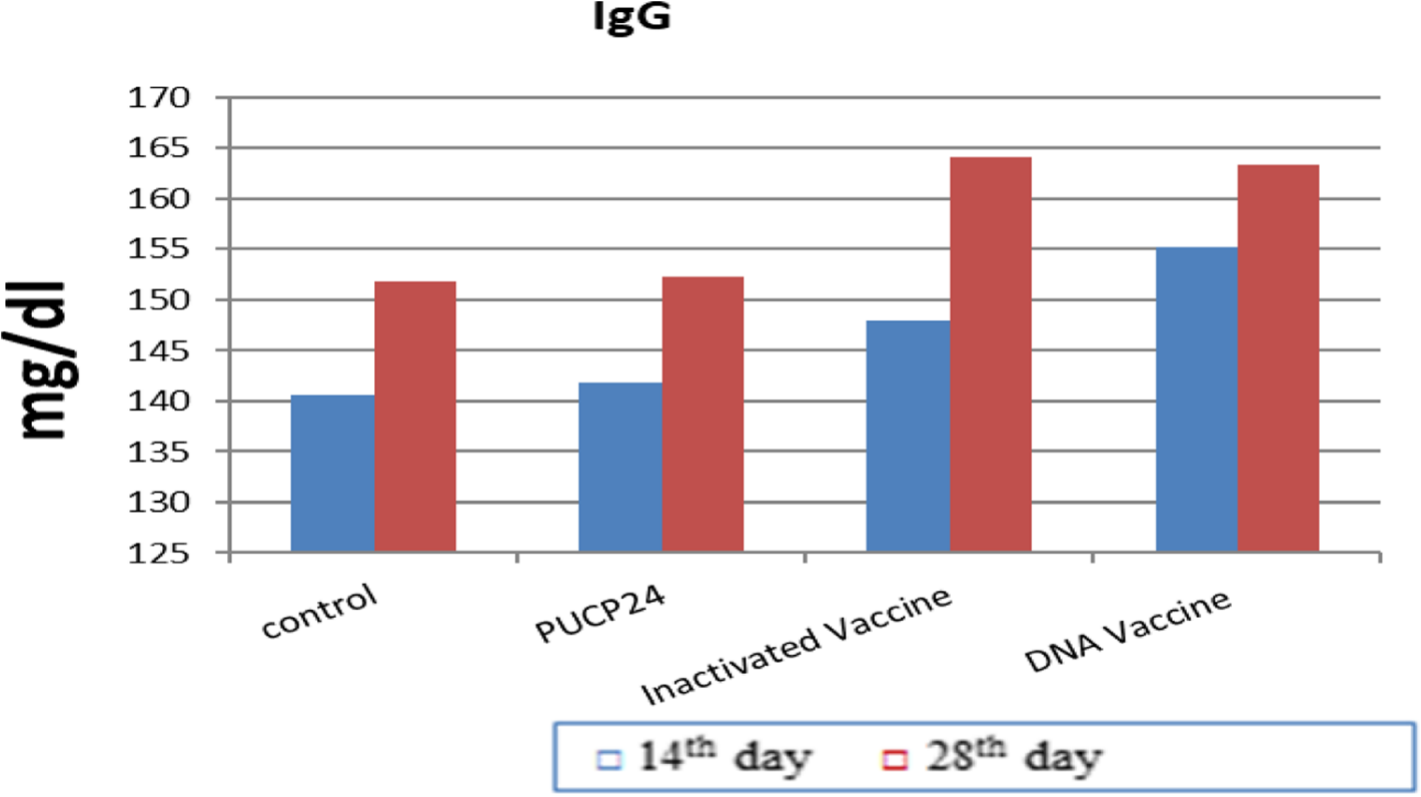
Serum IgG responses in immunized rats measured by indirect ELISA at the 14th day and 28th day of post-immunization. Each bar represents mean O.D. ± standard error of five pooled serum samples

The results of immune responses against inactivated and DNA vaccines (pUCP24-*OmpH*) in Fig. 6 shows that, after the first immunization of rats, the DNA vaccine group had higher significant levels (*P* < 0.05) of serum antibody IgM compared to the other groups (PBS, inactivated vaccine, and pUCP24 groups). In the second immunisation, the DNA vaccine group had the highest significant value of IgM, while the pUCP24 group had the lowest, despite the fact that DNA vaccine had a numerical increase in IgM value as compared to PBS and inactivated vaccine groups. In addition, there is a significant increase in IgG value of DNA vaccine after first immunization compared to the PBS and pUCP24 groups but not with the inactivated group as shown in Fig. 7, and IgG value of inactivated vaccine did not differ significantly with PBS and pUCP24 groups. However, after the second immunization, the DNA vaccine and inactivated vaccine groups had the highest values of IgG compared with PBS and pUCP24 groups.

**Fig. 8:**
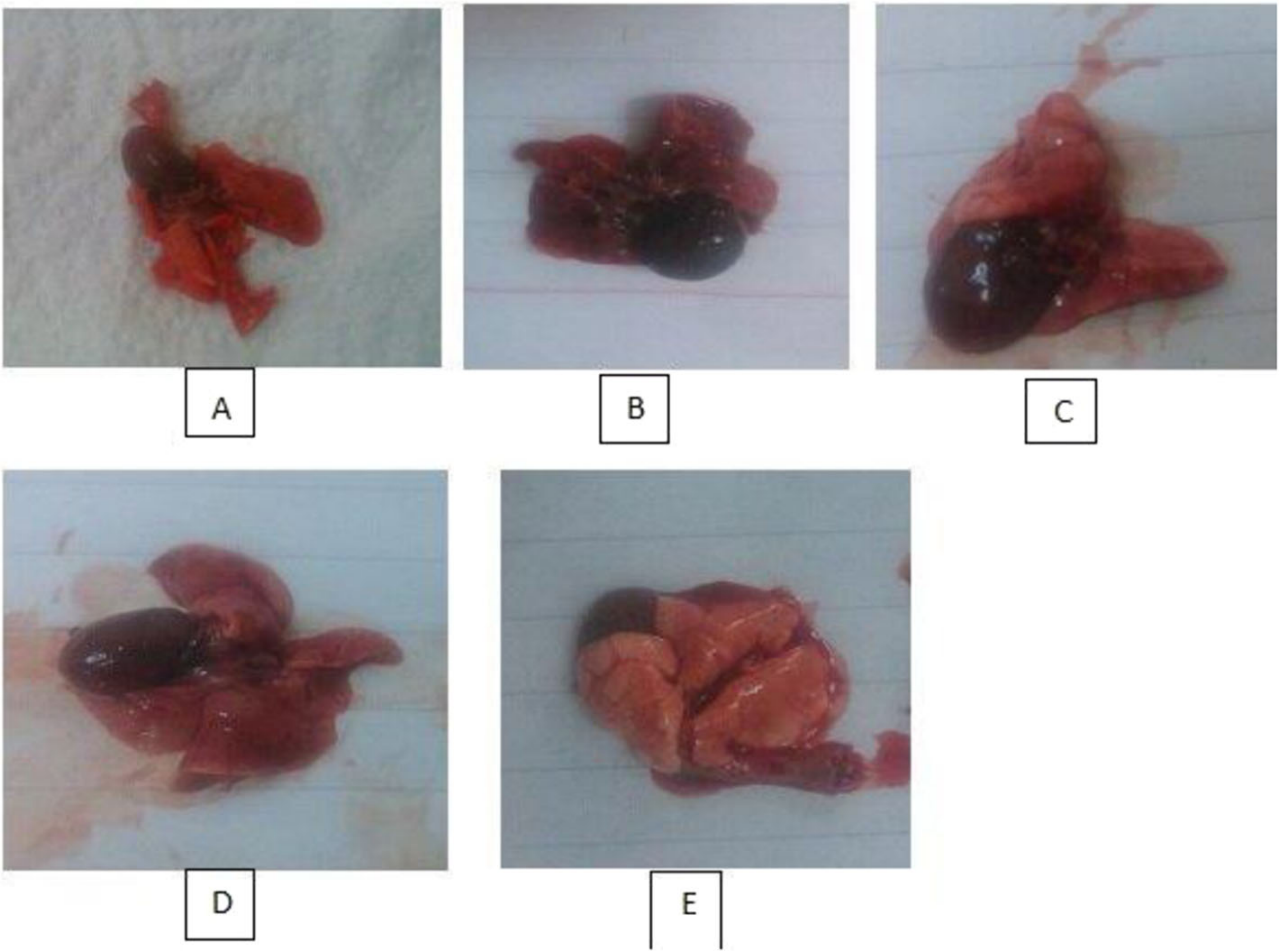
Photographic lesions in lungs of rat groups: **A** negative control, **B** PBS group, **C** pUCP24 group, **D** inactivated vaccine, and **E** DNA vaccine

Ten days after the last vaccination, four groups of rats were exposed to virulent *P. multocida* serotype A. The rats in the control group began to die the second day after the challenge. None of the rats in the control group survived more than 4 days after the challenge. No rats died in the DNA vaccine group or the inactivated vaccine group within 6 days after the challenge. Rats in the inactivated vaccine group began to die on the 7th day, and the number of surviving rats remained unchanged from the 8th day onwards. In the pUCP24 group, death occurred between days 4 and 7, and from then on, the number of surviving rats did not decrease further. Clinical signs manifested prior to death included weakness, inappetence, dyspnea, ataxia, and epistaxis. Postmortem findings recorded included congested lungs (Fig. 8). Frothy exudates were noticed in the lung of rats inoculated with *P. multocida* of serogroup A at a concentration of 10^8^ CFU. Rats in group 4 which were vaccinated with DNA vaccine exhibited clinical mortality.

### Vaccine efficacy

Groups of rats were challenged with virulent avian *P. multocida* serotype A at a concentration of 10^6^ CFU 10 days after the last immunization. Survival number and protection rate were counted until 14 days. The results in Table 1 show that the No. of death in pUCP24 and PBS groups was higher than the two DNA vaccines and the inactivated vaccine. However, the survival of rats immunized with inactivated vaccine (70%) was lower than those in the rats immunized with the DNA vaccine (90%). The mortality of rats injected with PBS was 100% after challenge whereas that of the pUCP24 group was 70%. In the DNA vaccine group, the relative protection rate was higher than in the inactivated group.

**Table 1:** Vaccine protective efficacy over 14 days in rats challenged with *Pasteurella multocida* Type A

Groups	Survival number/total rats	Mortality%
Group 1 (PBS)	0/10	100
Group 2 (inactivated vaccine)	6/10	40
Group 3 (pUCP24)	4/10	60
Group 4 (recombinant DNA vaccine)	9/10	10

## Discussion

Identification and characterization of important immunogens of the bacteria not only would help in designing an improved vaccine but also would help in developing the protection status of vaccinated animals. The OMPs of *P. multocida* are possible inducing immunogens for cattle [27]. Pati et al. [28] reported that OMP was immunogenic in buffalo calves and suggested that they could be used as a vaccine against hemorrhagic septicemia (HS). Many gram-negative bacteria have one or more predominant outer membrane proteins, and it has been shown that these proteins play an important role in host-pathogen interactions and disease processes such as *OmpA* and *OmpH* [29]. This study aimed to perform purification and expression of the major outer membrane gene (*OmpH*) of *P. multocida* strains isolated from Egypt and identified by Hassan et al. [22]. The results of PCR amplifying a product of the expected size of 1030 bp and a single PCR product with a similar molecular size were obtained from each strain reflecting the conserved nature of the *OmpH* gene among the *P. multocida* serotypes. The same results were reported by many researchers [30–33]. In addition, *OmpH* is highly conserved among *P. multocida* serotypes and is the only *P. multocida* protein where a small number of gene fragments are similar to eukaryotic cilia or flagella [11, 34].

The significance of PCR-RFLP types in the variation of virulence and other phenotypic characters among the *P. multocida* isolates remained to be elucidated [25]. In this research, PCR-RFLP is used for identifying the variability on the *OmpH* gene of *P. multocida*. Our results showed that *Bgl*II restriction endonuclease is useful for identifying variability of banding pattern and concluded that PCR-RFLP was a rapid test and could be done for selecting a different *OmpH* gene amplified from strain Nos. 2, 5, 7, and 10. The PCR analysis based on RFLP in *OmpH* gene is widely applied for the genetic classification of avian *P. multocida* isolated [30, 33] and for analyzing polymorphism within a gene segment [33, 35].

In this study, the *OmpH* gene of *P. multocida*, strain Nos. 2, 5, 7, and 10, was sequenced and analyzed. The sequence analysis indicated that *P. multocida* strains share maximal nucleotide identities (>90%) with avian *P. multocida* strains in the GenBank database in agreement with another study [32, 33, 36, 37]. Despite the similarity of the *OmpH* gene size amplified from the ten strains and the PCR-RFLP banding patterns of four strains, the phylogenetic tree showed the *OmpH* sequences of strain Nos. 2, 5, 7, and 10 were closed in the first cluster. Moreover, strain No. 10 was closed with two *P. multocida* strains KY403515 strain PM/VSVRI/2015 and KY403514 strain PM/VSVRI/2004; Abbas et al. [33] reported that the phylogenetic dendrogram of *OmpH* nucleotide sequence amplified from these strains showed that PM/VSVRI/2015 was clustered with PM/VSVRI/2004. The vaccine strain (PM/VSVRI/2004) was characterized and identified as PM type A and that used in FC vaccine®. Therefore, we select the *OmpH* gene amplified from strain No. 10 to construct a recombinant DNA vaccine.

Bacterial porin genes are sometimes difficult to clone in *E. coli* because external porins are usually lethal for *E. coli*. Initial attempts to clone the entire *OmpH* gene into the expression vector were unsuccessful by a number of workers [11, 15]. Non-succeed refers to the leaking expression of the primary protein without IPTG induction, and lethality of recombinant porin protein in *E. coli*. In this study, we cloned the *OmpH* gene into expression vector pUCP24, and the recombinant pUCP24-OmpH was expressed in *E. coli* DH5α. In similar studies, Luo et al. [15] cloned the *OmpH* gene of *P. multocida* X-73 into expression vector pQE30, and Singh et al. [38] cloned the PCR product of *OmpH* gene of *P. multocida* P52 into pQE32 expression vector, whereas the *P. multocida OmpH* was cloned in pET32a, and r*OmpH* was expressed in *E. coli* BLELISA (DE3) [32].

Immunogenicity and pathogenicity studies of various derivatives, components, and clones have been tested to find suitable immunization for *P. multocida* [39, 40]. Immunizations with genomic expression libraries has emerged as a novel technology [41, 42]. For identifying candidate vaccine genes that provide protection against pathogens, some studies have identified individual protective genes via the sequential fractionation of cDNA or genomic expression libraries [21, 43, 44]. The potential of *OmpH* gene as a vaccine candidate has been described and carried out in mice model against *P. multocida*. Thus, this study was conducted to analyze the immunogenicity from the pUCP24-OmpH recombinant clone. Sthitmatee et al. [45] suggested that serotyped *OmpH* proteins are strongly homologous and cross-protected. Other studies have also demonstrated that *OmpH* can induce high-level immune responses to homologous bacteria [35, 37, 46].

On the other hand, the serum obtained from vaccinated groups, positive control (killed bacterium), pUCP24 vector, and negative control (normal saline) provided results from ELISA showed positive results. ELISA was used to compare the immune response of mice to a *P. multocida* vaccine grown in the presence of serum to a splenocyte suspension [47]. The presence of IgG antibody from the serum of rats immunized with pUCP24-OmpH recombinant clone indicates a substantially higher antibody, thereby suggesting this DNA vaccine has the potential to induce a cellular immune response when compared to the negative control group. The experimental conformation was the intranasal administration of *P. multocida* in rats. The pathological study showed acute bronchopneumonia, which is in agreement with other studies [48, 49]. Luo et al. [15] vaccinated chickens with *P*. *multocida* serotype A:1 native and recombinant *OmpH*, and reported 100% and 18% safety, respectively. However, Sthitmatee et al. [45] showed that both the A:1 serotype native and recombinant *OmpH* have the same protein [13]; immunized mice with recombinant *OmpH* from *P*. *multocida* serotype B:2 causing hemorrhagic septicemia and obtained 100% protection against *P. multocida* at the same time as mice immunized subcutaneously, and challenged intraperitoneally only resulted in 80% protection. Moreover, recombinant *OmpH* from *P*. *multocida* (swine isolate) causing atrophic rhinitis resulted in 70% protection in mice [11]. Dabo et al. [17] used recombinant *OmpA* from a bovine isolate of *P*. *multocida* serotype A:3 to vaccinate mice, but no protection was obtained.

DNA vaccines are known as fourth-generation vaccines and were introduced to counter infection due to *Pasteurella* as it is cheaper compared to other types of vaccines in veterinary use [50]. There were few DNA vaccine against Pasteurellosis developed using different genes. DNA vaccine using *P. multocida* toxin (PMT) gene has the potential to protect an infected animal against infections caused by *P. multocida* [18].

## Conclusion

This study shows that the pUCP24-OmpH recombinant clone was successfully expressed and stable in *E. coli* DH5α host cells. We have shown that the DNA vaccine is capable of producing a high titer of antibody against *P. multocida*. Between all vaccinated rats, there is no lesion or inflammation detected and this may be used as a vaccine in the future.

## Data Availability

The data used and/or analyzed during the current study available from the corresponding author on reasonable request.

## Abbreviations

BLAST: Basic Local Alignment Search Tool
cDNA: Complementary DNA
CFU: Colony-forming unit
ELISA: Enzyme-linked immunosorbent assay
HS: Hemorrhagic septicemia
IgG3: Immunoglobulin G
IgM: Immunoglobulin M
NCBI: National Center for Biotechnology Information
*OmpH*: Outer membrane protein H
OMPs: Outer membrane proteins
PBS: Phosphate-buffered saline
PCR: Polymerase chain reaction
PMT: *P. multocida* toxin
RFLP: Restriction fragment length polymorphism
